# Correction: Visual stimulation with food pictures in the regulation of hunger hormones and nutrient deposition, a potential contributor to the obesity crisis

**DOI:** 10.1371/journal.pone.0236913

**Published:** 2020-07-23

**Authors:** The PLOS ONE Staff

Figs 1–5 are incorrect. The authors have provided corrected versions here. The publisher apologizes for the errors.

**Fig 1:**
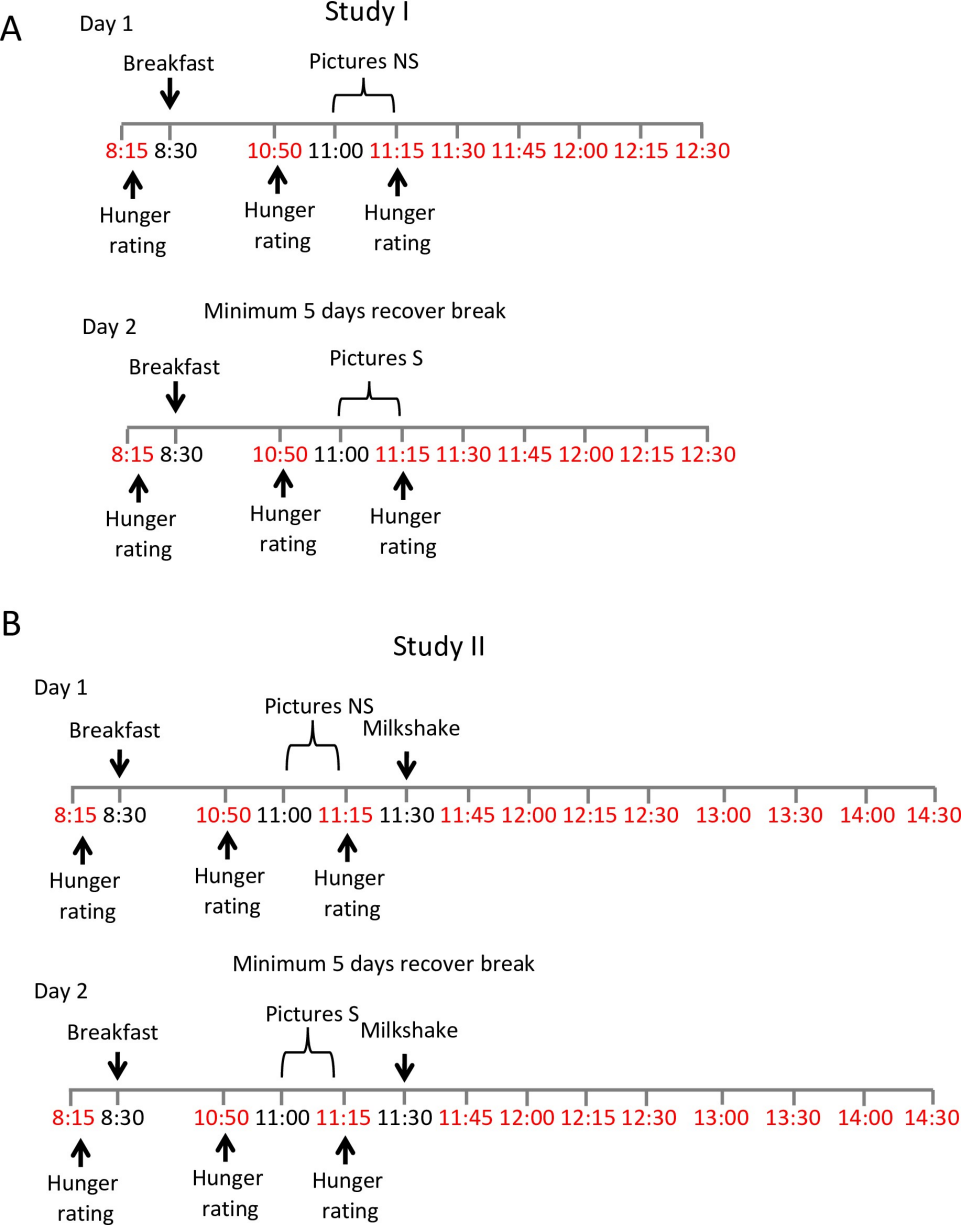
Schematic representation of the studies. During the study I (A) and study II (B) participants were given breakfast, asked to grade their hunger and to rate pictures of neutral objects or of food. For study II, following the picture session, the participants were given milkshake. The time points marked red indicate blood collection.

**Fig 2:**
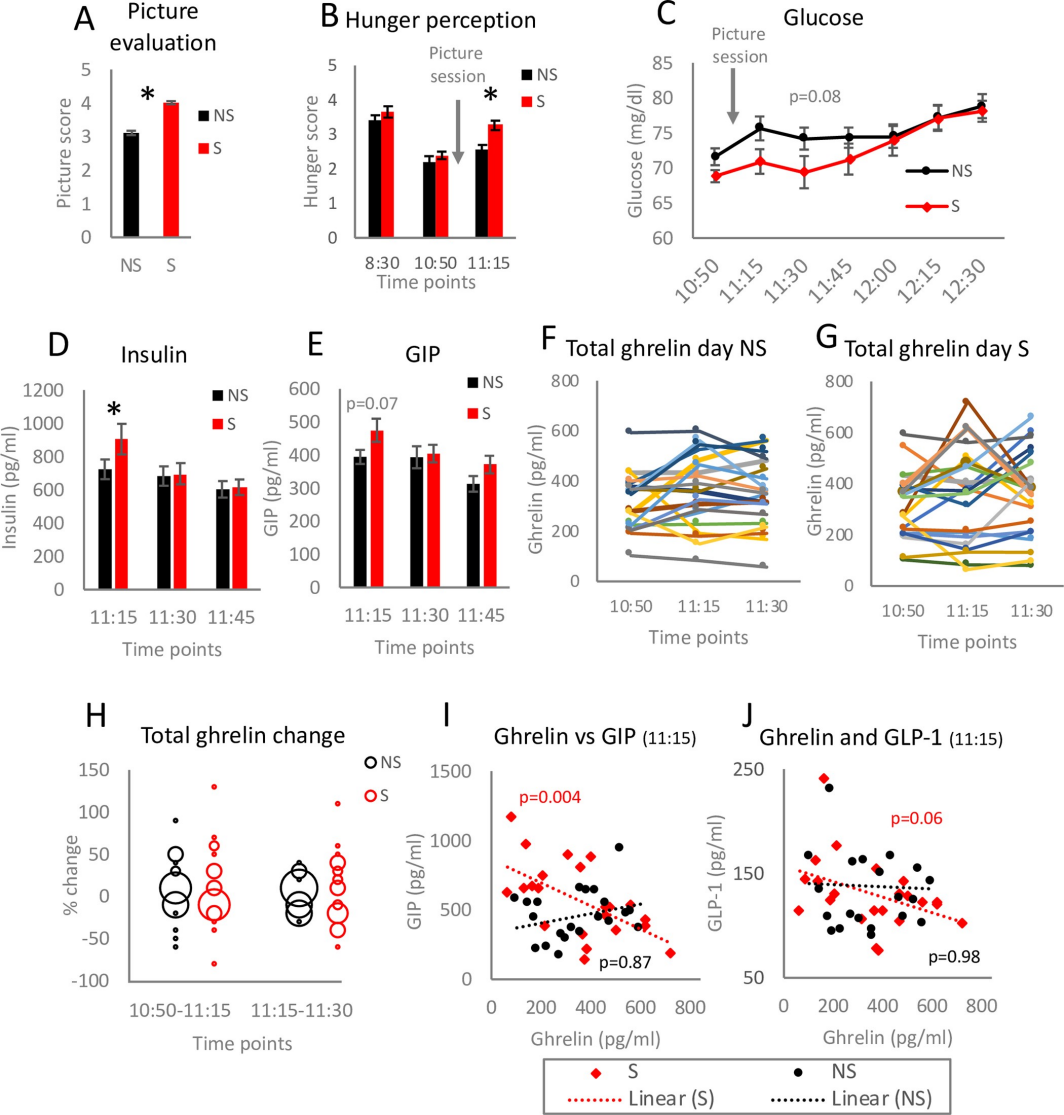
Food cues affect blood glucose and appetite-replated peptides. The participants rated different aspects of the pictures on a scale 1–5 (A). Hunger level was reported on a scale 1–5 by the participants at the indicated time points (B). Glucose levels were measured at all blood sampling time points (C). Insulin (D) and glucose-dependent insulinotropic peptide (GIP) (E) concentrations were quantified in blood collected at the indicated time points. For panel A statistical significance was analysed using two-tailed Student’s t-tests. Data (A-E) are presented as the mean±SEM; *p<0.05. For panels D-E ANOVA with Bonferroni correction for multiple testing was applied. Total ghrelin levels were measured in the samples collected on NS (F) and S (G) day of the study. Each line represents one participant. Distribution of total ghrelin concentration changes was evaluated by comparing % difference between two indicated time points (H). Linear regression analysis was performed to verify the correlation between ghrelin and GIP (I) as well as ghrelin and glucagon-like peptide-1 (GLP-1) (J) for blood samples collected at 11:15; n = 23.

**Fig 3:**
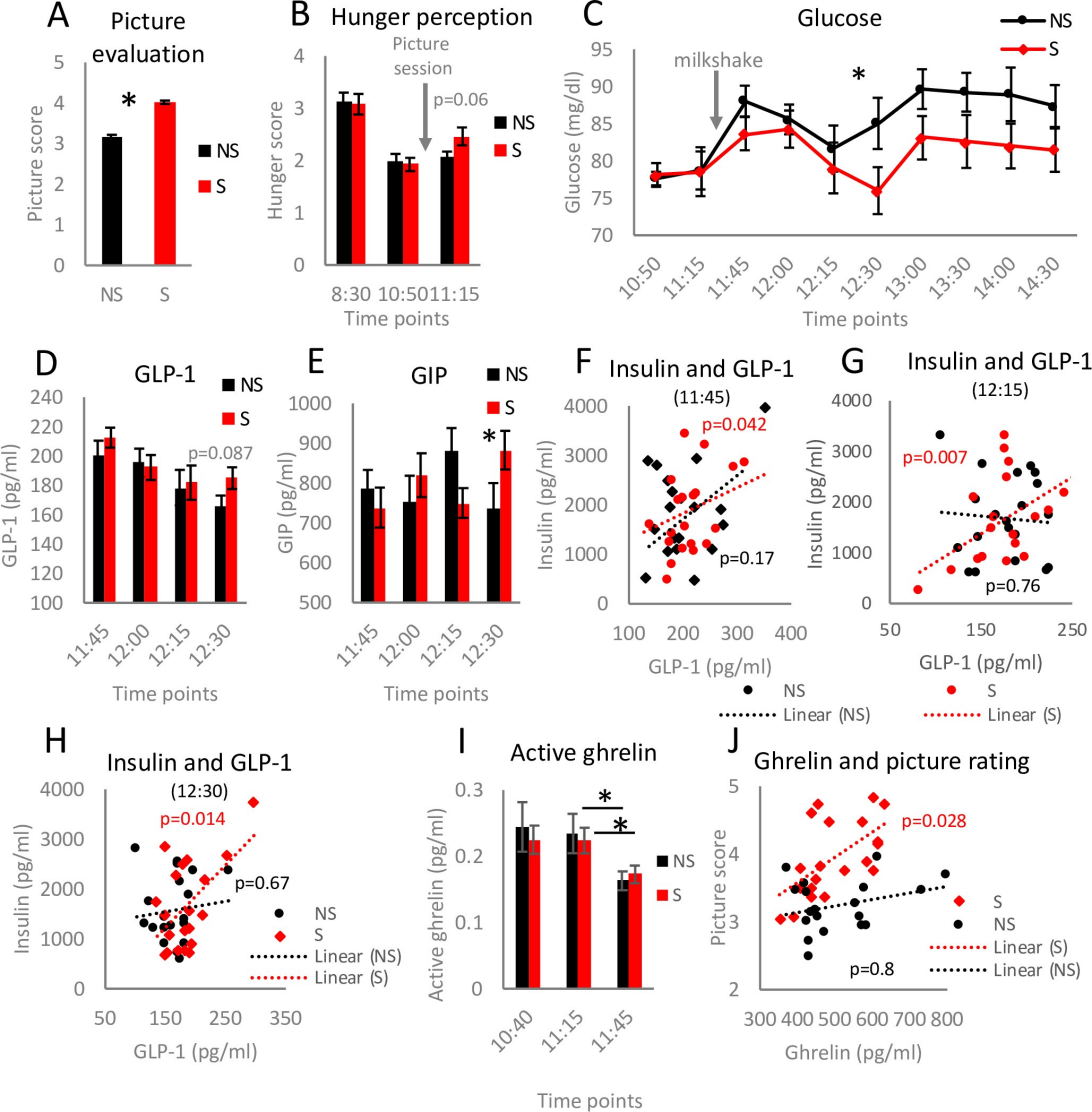
Exposure to food pictures influences postprandial blood glucose and appetite-related peptides. The participants ranked the pictures on a scale 1–5 (A). Hunger level on a scale 1–5 was rated by the participants at the indicated time points (B). Glucose levels were measured at the specified sampling time points (C). GIP (D) and GLP-1 (E) concentrations were quantified in blood samples collected at the indicated time points. Linear regression was used to validate the relationship between insulin and GLP-1 (F-H) for blood samples collected at the chosen time points. The concentration of active ghrelin in blood samples was assessed (I). Linear regression was applied to analyse interconnection between total ghrelin and picture score (J). Two-tailed Student’s t-tests were used to compare the experimental groups of panel A; n = 20, *p<0.05. For panels B, D, E and I ANOVA with Bonferroni correction for multiple testing was applied. Error bars represent the mean±SEM.

**Fig 4:**
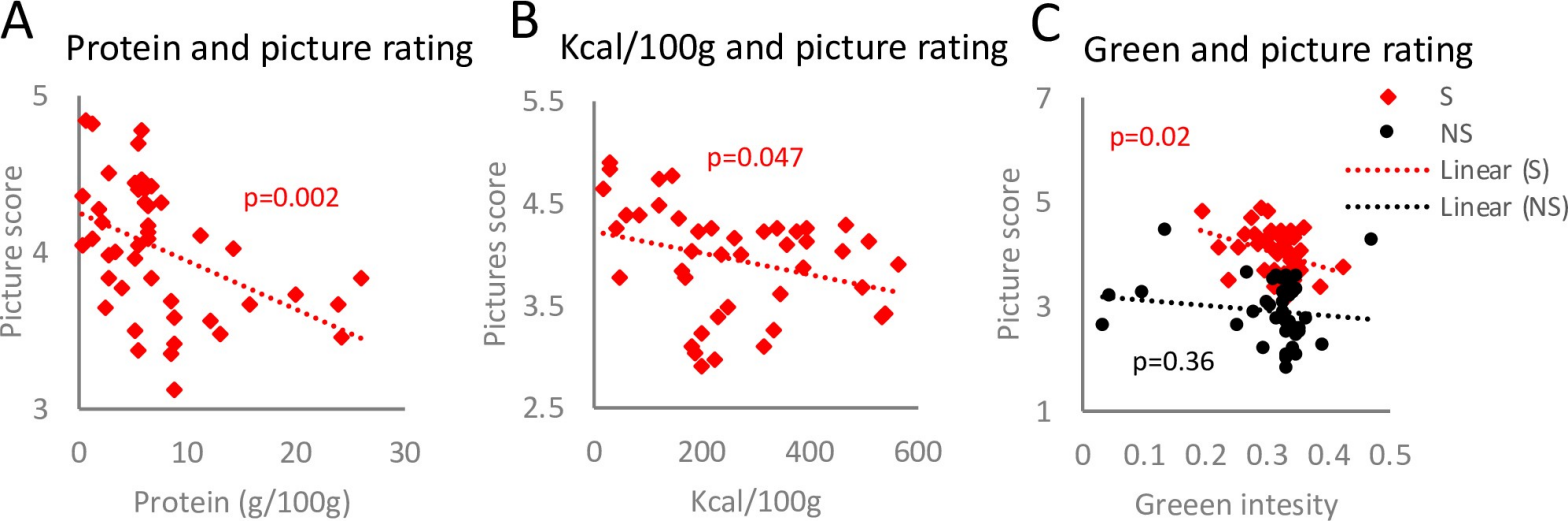
Participants’ preference towards pictures showing low protein, low calorie-dense foods with low green colour intensity. Linear regression was applied to analyse interconnection between pictured food protein content and corresponding rating (A), caloric content per 100g of the shown food and picture score (B) as well as the intensity of green colour and picture evaluation (C).

**Fig 5:**
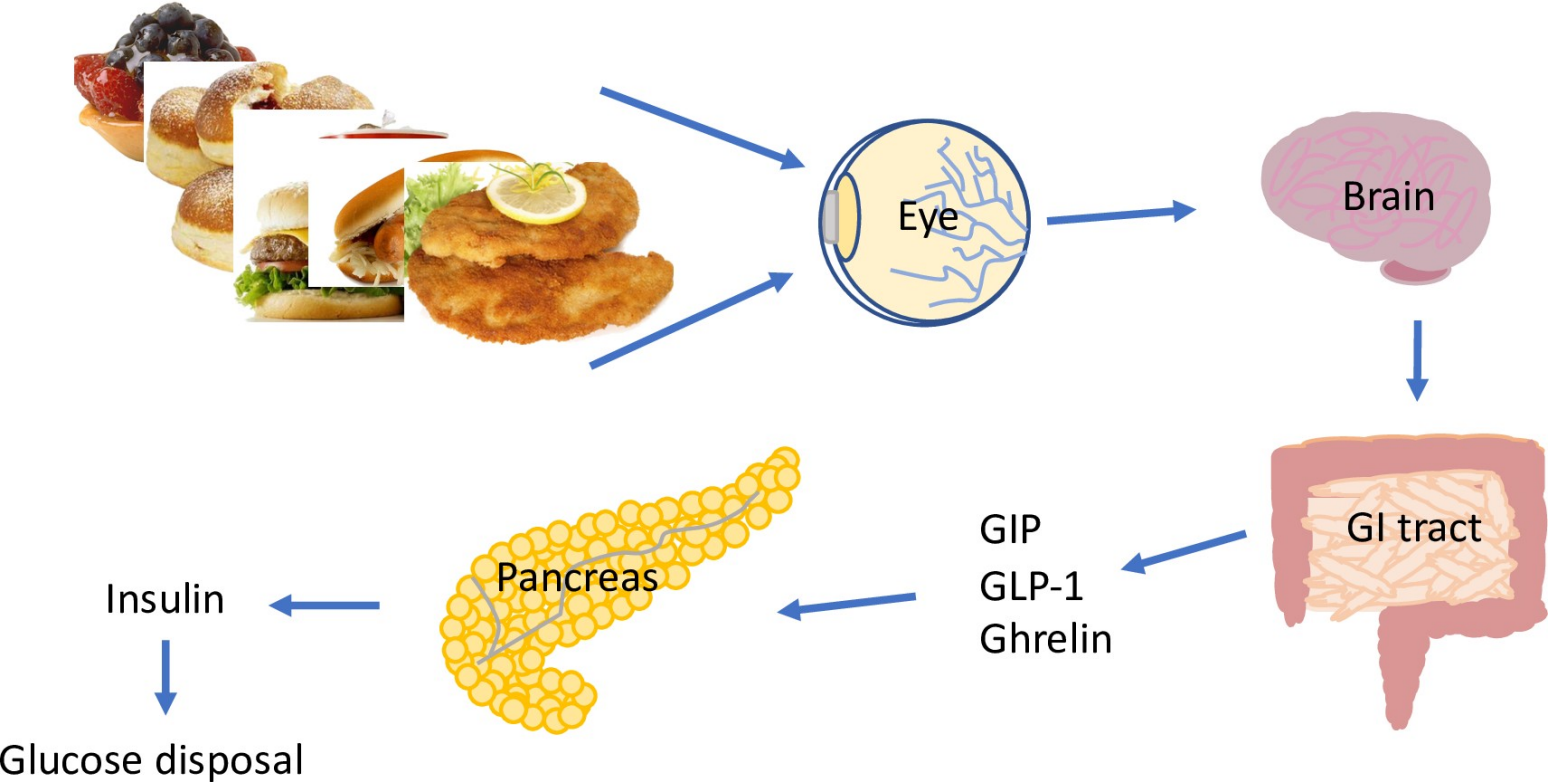
Schematic representation of the hypothesis. In study II, a small decrease in postprandial glucose concentration on S compared to NS day is observed for the first time point after the meal. Importantly, 1h after the meal, glucose concentrations drop further. This is most probably resulting from increased incretins and insulin concentrations and consequent glucose deposition. Interestingly, participants on the S day show delayed glucose decline and the most pronounced difference between NS and S day in glucose levels fits with the time point when the differences in GIP and GLP-1 concentrations between the two days of the study are the most pronounced. The differences between NS and S day imply that the efficacy of glucose uptake or deposition is affected by visual stimulation preceding a meal.
