# Supplementary material for: Visual stimulation with food pictures in the regulation of hunger hormones and nutrient deposition, a potential contributor to the obesity crisis
Source: PLoS One. 2020 Apr 24;15(4):e0232099. doi: 10.1371/journal.pone.0232099 (PMC7182185; doi:10.1371/journal.pone.0232099)
Supplement: S1 Table — Mean data are presented with±SD. (DOCX) [file pone.0232099.s001.docx]

|  | Study 1 | Study 2 |
| --- | --- | --- |
| Participants | 23 | 20 |
| Female | 20 | 19 |
| Male | 3 | 1 |
| Age | 23,43±2.9 | 23,05±3.1 |
| Body weight | 61,62±7.8 | 59,15±8.2 |
| BMI | 21,94±1.9 | 21,3±2.6 |

Supplementary table 1. Participants of study I and II. Mean data are presented with±SD.
