# Supplementary material for: Visual stimulation with food pictures in the regulation of hunger hormones and nutrient deposition, a potential contributor to the obesity crisis
Source: PLoS One. 2020 Apr 24;15(4):e0232099. doi: 10.1371/journal.pone.0232099 (PMC7182185; doi:10.1371/journal.pone.0232099)
Supplement: S2 Table — Food pictures presented a variety of products with indicated nutritional value and basic color intensity. The average participants’ answers to the 3 questions evaluating the pictures are shown for the Study I and II. (DOCX) [file pone.0232099.s002.docx]

|  | Nutrition (per 100g) | | | | Colour | | | Average picture score | |
| --- | --- | --- | --- | --- | --- | --- | --- | --- | --- |
| Object | Protein | Fat | Carb. | Kcal | Red | Green | Blue | Study 1 | Study 2 |
| chocolate cookie | 6,2 | 26 | 63 | 510 | 0,455 | 0,341 | 0,204 | 4,32 | 4,29 |
| snack mix | 9 | 0,5 | 75,3 | 347 | 0,484 | 0,329 | 0,187 | 3,58 | 3,48 |
| cheese burger, french fries and cola | 5,34 | 9 | 20,3 | 185 | 0,534 | 0,323 | 0,143 | 3,50 | 3,09 |
| German krapfen | 6,67 | 13,33 | 41,67 | 317 | 0,449 | 0,335 | 0,215 | 4,43 | 4,41 |
| chips | 5,48 | 35 | 48 | 539 | 0,505 | 0,385 | 0,110 | 3,38 | 3,64 |
| apple pie | 2,9 | 7,8 | 37,4 | 237 | 0,466 | 0,335 | 0,199 | 3,85 | 4,26 |
| pizza (salami) | 6,55 | 5,64 | 21,27 | 165,45 | 0,597 | 0,254 | 0,149 | 4,10 | 3,93 |
| doner kebab | 12,11 | 4,91 | 24,31 | 192 | 0,518 | 0,312 | 0,170 | 3,57 | 3,04 |
| cheese platter | 24 | 29 | 0,05 | 357 | 0,413 | 0,355 | 0,232 | 3,67 | 3,68 |
| raspberry cake | 3,5 | 12 | 34,3 | 262 | 0,394 | 0,317 | 0,289 | 4,00 | 4,22 |
| Sacher cake | 5,4 | 15,4 | 51,5 | 375 | 0,532 | 0,263 | 0,204 | 4,40 | 4,33 |
| sundae (with raspberries) | 1,9 | 12,37 | 15,9 | 198 | 0,480 | 0,301 | 0,218 | 4,28 | 4,22 |
| pastries and donuts | 6,4 | 22,59 | 39,78 | 390 | 0,481 | 0,328 | 0,191 | 4,13 | 4,00 |
| chocolate muffin | 6,2 | 20,7 | 49 | 398 | 0,487 | 0,340 | 0,172 | 4,40 | 4,42 |
| pasta bake | 5,7 | 5,1 | 13 | 123 | 0,532 | 0,304 | 0,164 | 4,47 | 4,67 |
| round shortbread cookies | 6,7 | 29 | 62 | 536 | 0,483 | 0,348 | 0,169 | 3,83 | 3,67 |
| popcorn | 7,7 | 15,2 | 53,3 | 397 | 0,359 | 0,335 | 0,305 | 4,32 | 4,36 |
| peanut puffs | 13 | 24 | 56 | 500 | 0,502 | 0,344 | 0,155 | 3,48 | 3,80 |
| cookies filled with chocolate cream | 5,5 | 16,5 | 71,8 | 460 | 0,524 | 0,308 | 0,168 | 4,05 | 4,16 |
| marble cake | 5,3 | 22 | 48,3 | 423 | 0,493 | 0,323 | 0,184 | 4,45 | 4,41 |
| bowl of muesli (granola) | 9 | 6,04 | 59,6 | 343 | 0,471 | 0,328 | 0,202 | 3,42 | 4,04 |
| watermelon | 0,26 | 0,09 | 3,64 | 17 | 0,572 | 0,279 | 0,149 | 4,37 | 4,58 |
| raspberries | 1,2 | 0,3 | 4,6 | 32 | 0,672 | 0,192 | 0,136 | 4,82 | 4,87 |
| strawberries | 0,78 | 0,39 | 5,33 | 31 | 0,581 | 0,292 | 0,127 | 4,85 | 4,84 |
| figs | 1,29 | 0,49 | 12,77 | 62 | 0,587 | 0,222 | 0,191 | 4,10 | 4,35 |
| sushi | 5,2 | 1,7 | 28,9 | 162 | 0,379 | 0,346 | 0,274 | 3,97 | 4,07 |
| chocolate cookies | 5,5 | 21,1 | 67,3 | 471,1 | 0,513 | 0,276 | 0,212 | 4,70 | 4,51 |
| salmon | 20 | 9,57 | 1,3 | 170 | 0,453 | 0,342 | 0,205 | 3,73 | 3,80 |
| Potatoe Wedges | 2,7 | 2,2 | 25,5 | 123 | 0,487 | 0,356 | 0,157 | 4,52 | 4,72 |
| Hot Dog | 8,92 | 9,57 | 26,23 | 226,4 | 0,525 | 0,324 | 0,151 | 3,13 | 2,91 |
| blueberry a. strawberry tartlet | 2,12 | 6,43 | 18,4 | 217,54 | 0,508 | 0,285 | 0,207 | 4,20 | 4,17 |
| bagels with sesame and poppy seed | 11,38 | 3,92 | 50,4 | 276,1 | 0,492 | 0,323 | 0,186 | 4,12 | 4,09 |
| goose, roasted | 15,7 | 31 | 0 | 338 | 0,447 | 0,311 | 0,242 | 3,67 | 3,59 |
| pears | 0,5 | 0,3 | 12,4 | 52 | 0,490 | 0,355 | 0,155 | 4,05 | 3,99 |
| cream roll | 2,4 | 15,2 | 25,2 | 248 | 0,488 | 0,325 | 0,187 | 3,65 | 3,55 |
| pralines | 6,5 | 38 | 50 | 566 | 0,474 | 0,298 | 0,228 | 4,17 | 4,00 |
| pancake with fruits | 5,7 | 7,3 | 14,2 | 148 | 0,453 | 0,301 | 0,246 | 4,78 | 4,83 |
| burger patty with french fries and salad | 8,45 | 10,2 | 20,87 | 199,64 | 0,464 | 0,311 | 0,224 | 3,35 | 3,04 |
| vanilla and chocolate icecream cone | 2,7 | 8,6 | 23,5 | 182 | 0,497 | 0,339 | 0,164 | 3,98 | 3,97 |
| spare ribs with french fries and salad | 8,63 | 11,26 | 18,56 | 202,14 | 0,492 | 0,293 | 0,216 | 3,68 | 3,48 |
| walnut | 14,4 | 62,5 | 10,6 | 654 | 0,545 | 0,329 | 0,126 | 4,03 | 3,97 |
| mixed vegetables | 4 | 0,4 | 5,5 | 43 | 0,459 | 0,421 | 0,120 | 3,77 | 4,00 |
| Viennese Schnitzel | 26,1 | 9,3 | 6,5 | 230 | 0,510 | 0,345 | 0,146 | 3,85 | 3,58 |
| tomato and mozzarella | 6,5 | 5,3 | 2 | 86,7 | 0,537 | 0,320 | 0,143 | 4,30 | 4,35 |
| salami, roasted ham and cheese | 24,32 | 25,5 | 1,7 | 318,5 | 0,615 | 0,235 | 0,150 | 3,47 | 3,13 |

Supplementary table 2. Pictures presented on day S of the study. Food pictures presented a variety of products with indicated nutritional value and basic color intensity. The average participants’ answers to the 3 questions evaluating the pictures are shown for the Study I and II.
