# Supplementary material for: Visual stimulation with food pictures in the regulation of hunger hormones and nutrient deposition, a potential contributor to the obesity crisis
Source: PLoS One. 2020 Apr 24;15(4):e0232099. doi: 10.1371/journal.pone.0232099 (PMC7182185; doi:10.1371/journal.pone.0232099)
Supplement: S3 Table — Pictures presented a variety of objects with indicated basic color intensity. The average participants’ answers to the 3 questions evaluating the pictures are shown for the Study I and II. (DOCX) [file pone.0232099.s003.docx]

|  | Colours | | | Average picture score | |
| --- | --- | --- | --- | --- | --- |
| Object | Red | Green | Blue | Study 1 | Study 2 |
| flower | 0,796 | 0,137 | 0,068 | 4,43 | 4,52 |
| blue gemstone | 0,103 | 0,339 | 0,558 | 3,24 | 3,32 |
| red bucket | 0,891 | 0,033 | 0,077 | 2,63 | 2,48 |
| light bulb | 0,340 | 0,324 | 0,336 | 2,71 | 2,91 |
| hammer | 0,495 | 0,348 | 0,158 | 2,06 | 2,35 |
| clothes hanger | 0,404 | 0,338 | 0,257 | 2,70 | 2,88 |
| ladder | 0,414 | 0,335 | 0,251 | 2,56 | 3,00 |
| nails | 0,357 | 0,332 | 0,311 | 1,81 | 2,13 |
| paintbrushes | 0,338 | 0,340 | 0,322 | 2,57 | 3,12 |
| thumbtacks | 0,453 | 0,341 | 0,207 | 2,19 | 2,04 |
| scissors | 0,335 | 0,334 | 0,331 | 2,02 | 2,54 |
| stone (tower) | 0,337 | 0,328 | 0,335 | 3,48 | 3,80 |
| ballpen | 0,270 | 0,318 | 0,412 | 2,71 | 3,03 |
| tacker | 0,348 | 0,332 | 0,319 | 2,03 | 2,54 |
| pocketknife | 0,422 | 0,297 | 0,281 | 2,21 | 2,30 |
| telephone | 0,333 | 0,333 | 0,333 | 2,67 | 3,04 |
| tree leaf | 0,641 | 0,267 | 0,092 | 3,65 | 4,22 |
| books | 0,342 | 0,339 | 0,319 | 3,43 | 3,55 |
| sponge | 0,353 | 0,392 | 0,255 | 2,25 | 2,32 |
| flower bouquet | 0,424 | 0,471 | 0,105 | 4,25 | 4,65 |
| hair brush | 0,440 | 0,305 | 0,256 | 3,00 | 3,10 |
| contact lense clensing fluid | 0,295 | 0,351 | 0,354 | 2,52 | 3,20 |
| brown stones | 0,435 | 0,329 | 0,236 | 3,27 | 3,52 |
| chess men | 0,362 | 0,328 | 0,310 | 3,05 | 3,46 |
| shelf | 0,349 | 0,336 | 0,316 | 3,19 | 3,45 |
| football | 0,512 | 0,254 | 0,234 | 2,60 | 3,03 |
| checkerboard | 0,364 | 0,336 | 0,300 | 2,56 | 3,06 |
| shopping bag | 0,805 | 0,096 | 0,099 | 3,27 | 3,09 |
| Bleach (packaging) | 0,323 | 0,278 | 0,399 | 2,87 | 2,74 |
| yellow lunch box | 0,447 | 0,347 | 0,206 | 3,29 | 3,16 |
| brown wallet | 0,439 | 0,299 | 0,262 | 3,08 | 3,19 |
| gift wrapped in brown paper | 0,401 | 0,339 | 0,260 | 3,41 | 3,84 |
| handkerchiefs | 0,171 | 0,333 | 0,496 | 3,56 | 3,45 |
| hand shovel | 0,303 | 0,346 | 0,351 | 2,44 | 2,45 |
| screw and nuts | 0,451 | 0,348 | 0,201 | 2,56 | 2,46 |
| tape | 0,357 | 0,340 | 0,303 | 3,22 | 3,30 |
| calculator | 0,367 | 0,354 | 0,279 | 2,49 | 2,26 |
| paper clips | 0,365 | 0,330 | 0,305 | 2,46 | 2,64 |
| butterfly | 0,460 | 0,348 | 0,192 | 3,54 | 3,72 |
| chair | 0,053 | 0,315 | 0,631 | 3,59 | 3,70 |
| pocket watch | 0,433 | 0,344 | 0,222 | 3,27 | 3,51 |
| umbrella | 0,878 | 0,042 | 0,080 | 3,19 | 3,20 |
| spool of thread | 0,436 | 0,326 | 0,238 | 2,86 | 3,09 |
| flash light / torch | 0,419 | 0,362 | 0,219 | 2,71 | 2,74 |
| hot-air ballon | 0,433 | 0,309 | 0,257 | 3,49 | 3,84 |

Supplementary table 3. Pictures presented on day NS of the study. Pictures presented a variety of objects with indicated basic color intensity. The average participants’ answers to the 3 questions evaluating the pictures are shown for the Study I and II.
