# Supplementary material for: Visual stimulation with food pictures in the regulation of hunger hormones and nutrient deposition, a potential contributor to the obesity crisis
Source: PLoS One. 2020 Apr 24;15(4):e0232099. doi: 10.1371/journal.pone.0232099 (PMC7182185; doi:10.1371/journal.pone.0232099)
Supplement: S4 Table — The data are presented with±SEM. (DOCX) [file pone.0232099.s004.docx]

| Time point | 11:15 | | 11:30 | | 11:45 | |
| --- | --- | --- | --- | --- | --- | --- |
| Day  pg/ml | NS | S | NS | S | NS | S |
| GIP | 394,5±21 | 475,1±35 | 393,5±33 | 404,6±27 | 313,1±24 | 371,4±27 |
| GLP-1 | 138,1±8,6 | 129,8±7 | 132,7±8,3 | 125,9±5,9 | 135,4±8,5 | 134,8±6,9 |
| PYY | 152,9±11,3 | 145,8±6,3 | 148,2±8,3 | 143,7±7,1 | 145,1±7,2 | 142,7±5,4 |
| Insulin | 722,8±59 | 904±92 | 682,4±58 | 692,4±64 | 602,6±49 | 615,3±47 |
| Glucagon | 107,9±4,4 | 103,8±2 | 105,3±2,9 | 101,4±2,3 | 105,7±2,8 | 102,2±1,6 |

Supplementary table 4. Blood concentration of hunger-associated peptides in study I. The data are presented with±SEM.
