# Supplementary material for: Visual stimulation with food pictures in the regulation of hunger hormones and nutrient deposition, a potential contributor to the obesity crisis
Source: PLoS One. 2020 Apr 24;15(4):e0232099. doi: 10.1371/journal.pone.0232099 (PMC7182185; doi:10.1371/journal.pone.0232099)
Supplement: S5 Table — The data are shown as±SEM. (DOCX) [file pone.0232099.s005.docx]

| Time point | 11:45 | | 12:00 | | 12:15 | | 12:30 | |
| --- | --- | --- | --- | --- | --- | --- | --- | --- |
| Day  pg/ml | NS | S | NS | S | NS | S | NS | S |
| GIP | 783,87±49 | 738,58±50 | 753,25±66 | 819,89±55 | 878,24±60 | 750,19±37 | 737,04±63 | 882,81±48 |
| GLP-1 | 200±9,5 | 212,51±6,8 | 195,15±9,5 | 192,15±8,4 | 178,4±12 | 181,84±11 | 165,13±7,9 | 184,9±7,4 |
| PYY | 131,67±20,9 | 129,42±21,7 | 135,92±18,6 | 130,1±17,2 | 128,64±19,8 | 125,3±24,4 | 125,99±22,7 | 123,86±21,8 |
| Insulin | 1832,33±128 | 1824,32±210 | 2043,69±199 | 2073 ,49±197 | 1676,84±197 | 1729,45±211 | 1574,6±184 | 1665,01±152 |
| Glucagon | 93,92±6,1 | 106,69±6,2 | 97,55±6 | 105,53±5,5 | 94,2±5,3 | 98,95±7,3 | 89,58±6,5 | 97,79±5,9 |

Supplementary table 5. Blood concentration of hunger peptides in study II. The data are shown as±SEM.
