# Supplementary figures and images for: Visual stimulation with food pictures in the regulation of hunger hormones and nutrient deposition, a potential contributor to the obesity crisis
Source: PLoS One. 2020 Apr 24;15(4):e0232099. doi: 10.1371/journal.pone.0232099 (PMC7182185; doi:10.1371/journal.pone.0232099)

## Supplementary data

### Supplementary figure 1

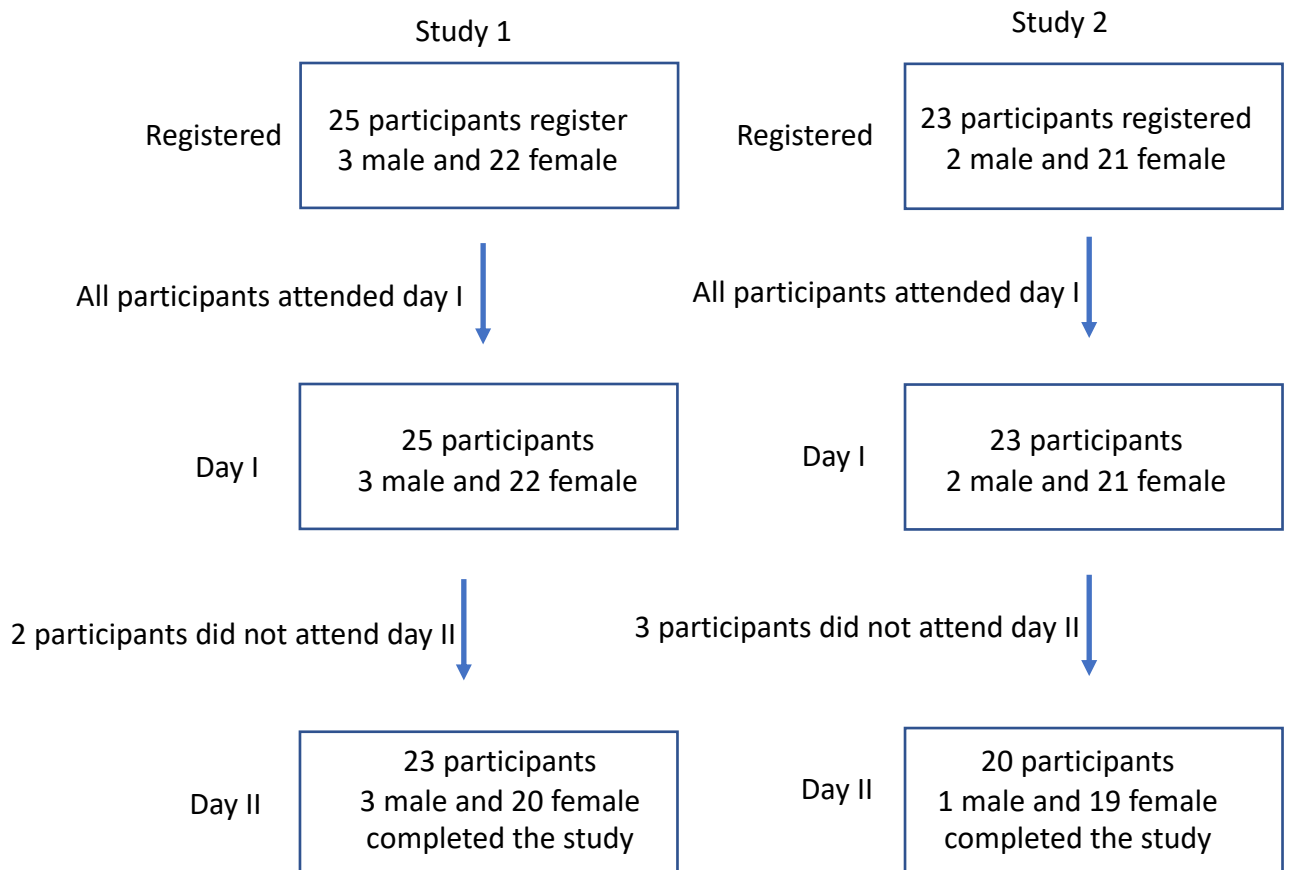

Supplement: S1 Fig — (PDF) [file pone.0232099.s006.pdf]

# Supplementary data

## Supplementary figure 2

D

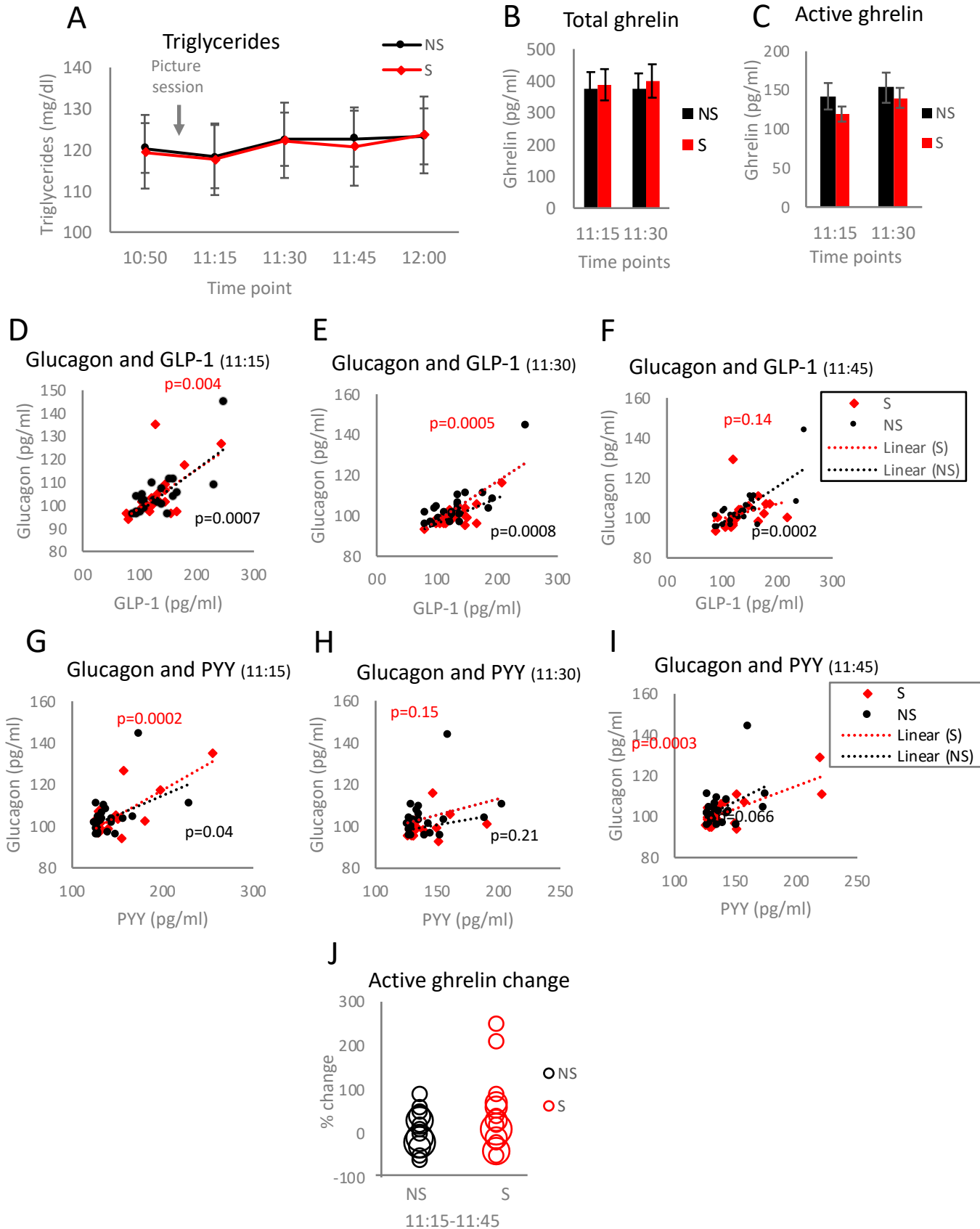

Supplement: S2 Fig — Concentration of glucagon and GLP-1 as well as glucagon and PYY show correlation. Blood levels of triglycerides were measured between 10:50 and 12:00 on both days of the study (A). The concentration of total (B) and active (C) ghrelin were measured in the collected blood samples. ANOVA with Bonferroni correction for multiple testing was used to assess statistical differences. Data (A-C) are presented as the mean±SEM. Linear regression was analyzed to verify the connection between glucagon and GLP-1 (D-F) as well as glucagon and PYY (G-I) for samples collected at the indicated time points. Changes of the concentration of active ghrelin were calculated (J); n = 23. (PDF) [file pone.0232099.s007.pdf]

## Supplementary data

### Supplementary figure 3

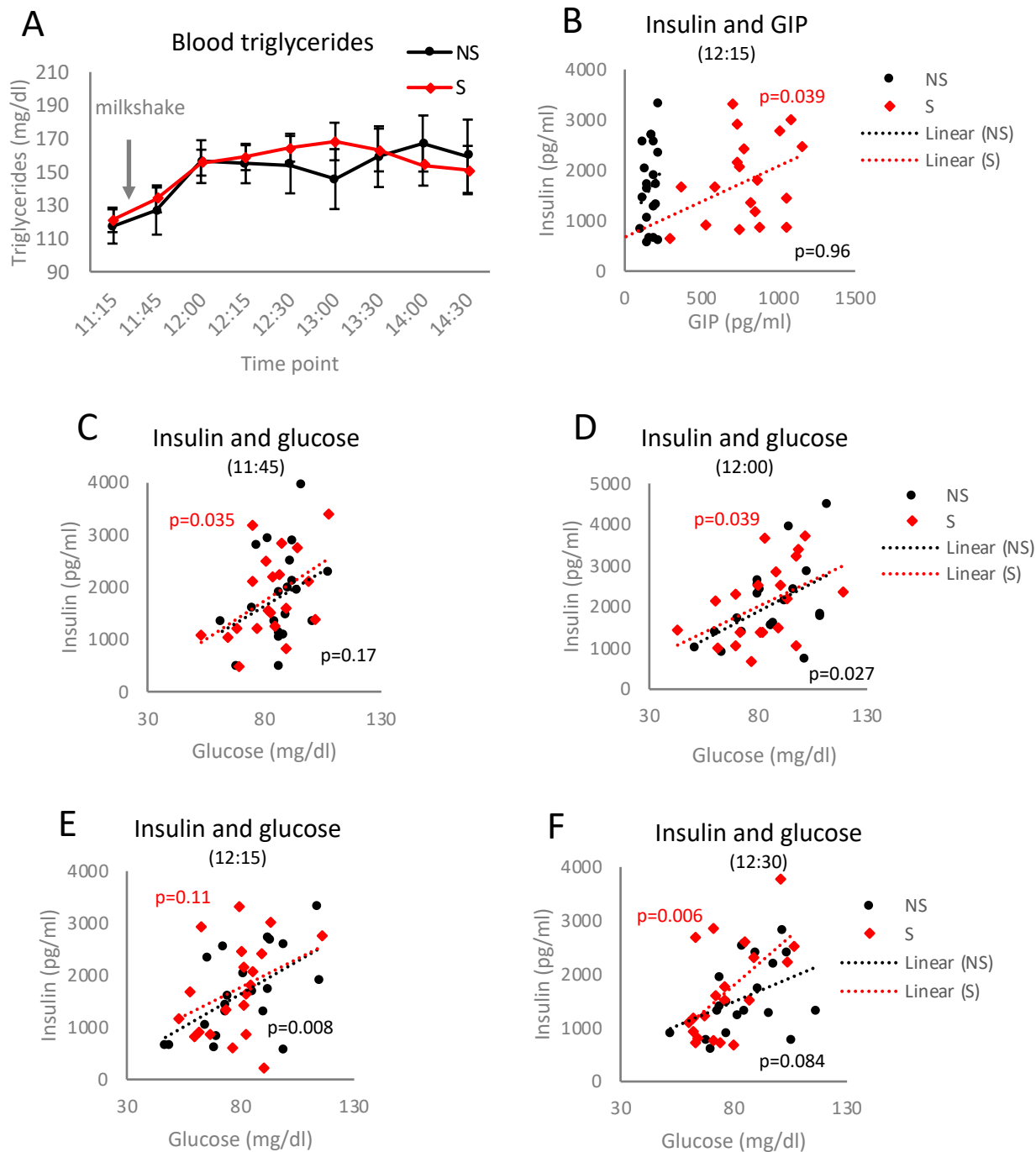

Supplement: S3 Fig — Blood levels of triglycerides were measured between 11:15 and 14:30 on both days of the study (A). Data are presented as the mean±SEM. Linear regression was analysed for correlation between insulin and GIP (B) as well as insulin and glucose for samples collected at 11:45 (C), 12:00 (D), 12:15 (E) and 12:30 (F); n = 20. (PDF) [file pone.0232099.s008.pdf]

# Supplementary data

## Supplementary figure 4

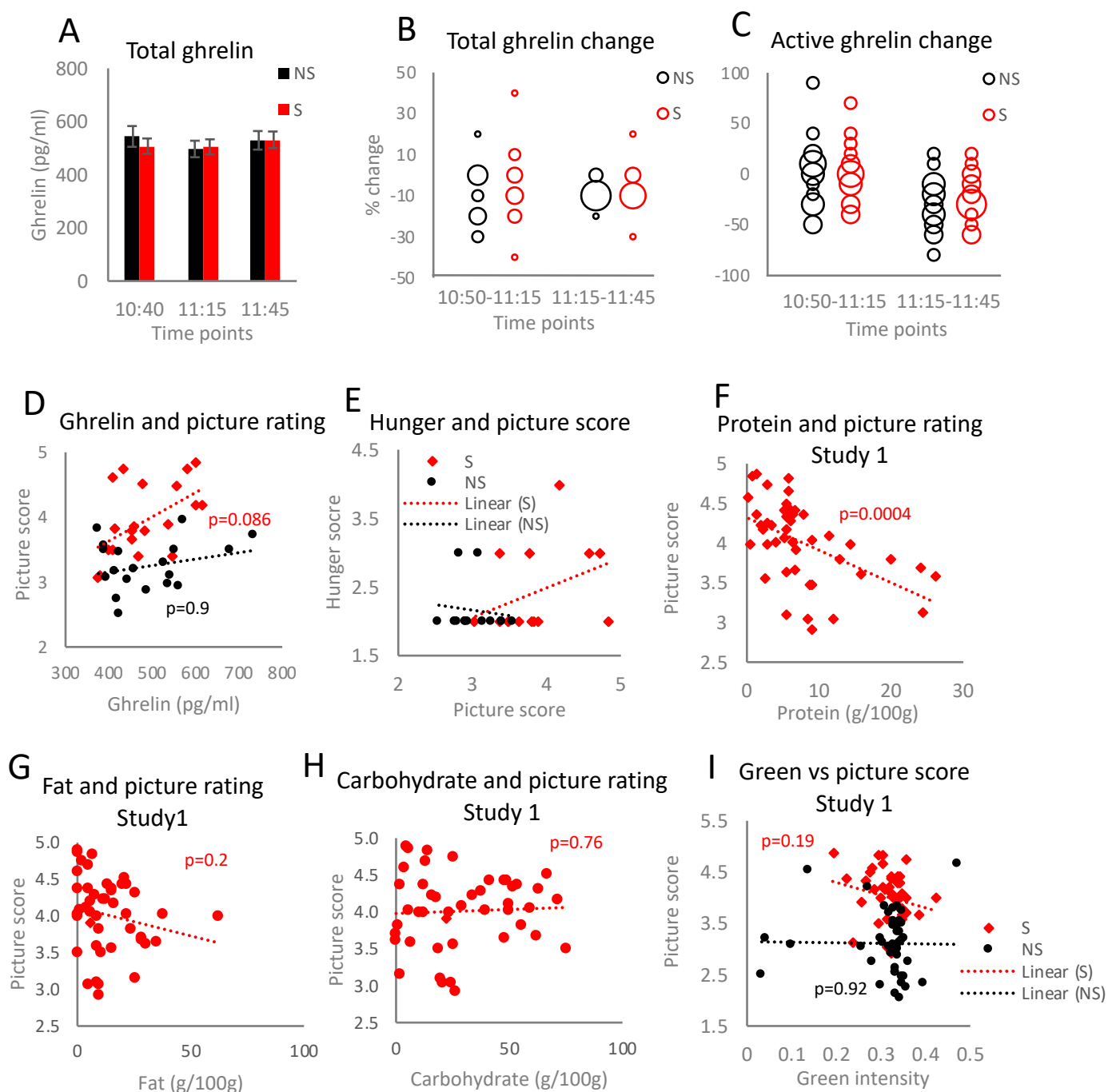

Supplement: S4 Fig — Nutritional as well as visual features of the food pictures influence attractiveness of the food pictures. The concentration of total ghrelin was measured in the blood samples collected at the indicated time points (A). Distribution of the concentration of total (B) and active (C) ghrelin changes were evaluated by comparing % difference between two indicated time points. Linear regression analysis was performed to verify the correlation between total ghrelin and picture evaluation score (D). Linear regression was analyzed for the relationship between hunger rating and picture evaluation score (E), food protein content (F), fat content (E), carbohydrate content. (H) and picture evaluation score as well as green color intensity and picture evaluation score (I). ANOVA with Bonferroni correction for multiple testing was used to determine statistical significance for the data in panels A, E and F; n = 20, *p<0.05. (PDF) [file pone.0232099.s009.pdf]

Supplementary data

Supplementary figure 5

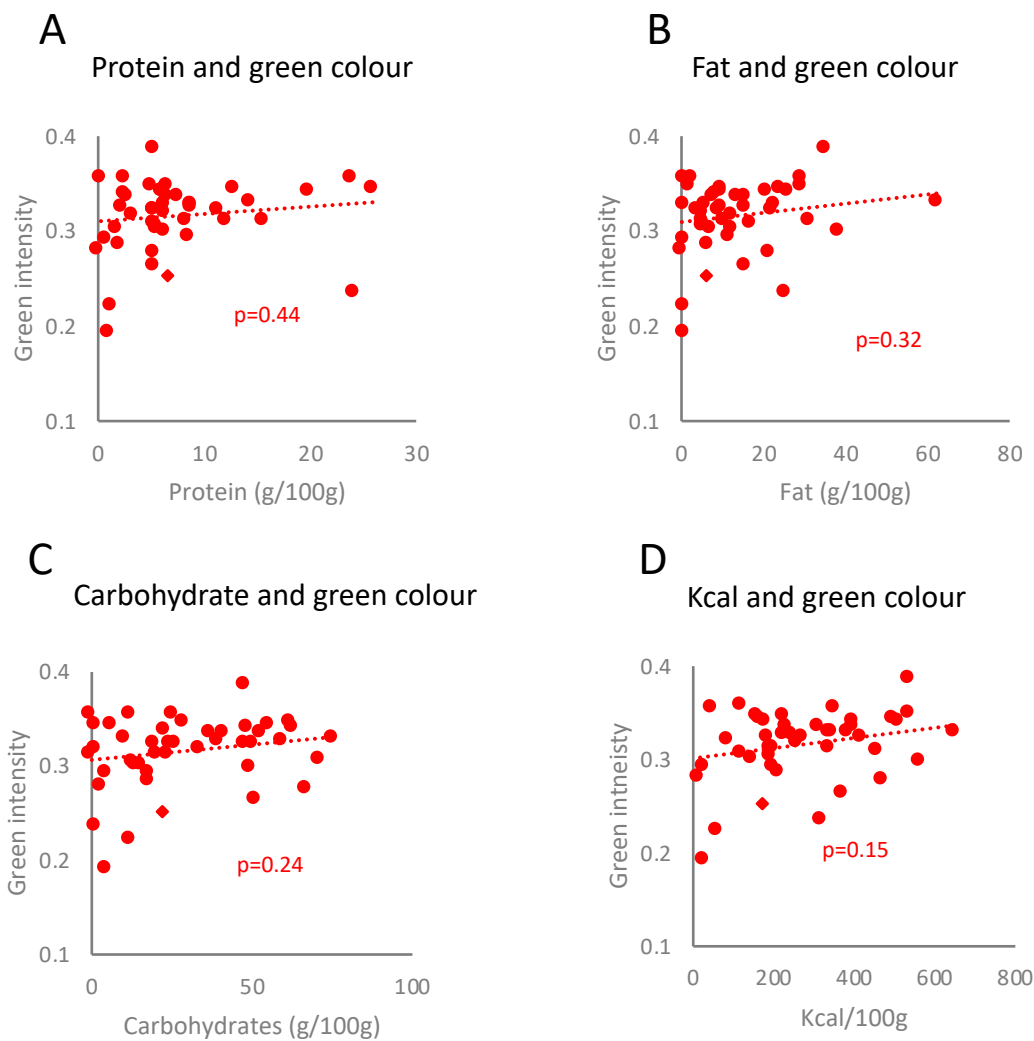

Supplement: S5 Fig — Linear regression was analyzed for the relationship between protein (A), fat (B), carbohydrate (C), caloric density (D) versus intensity of green colour. study II. The data are shown as±SEM. (PDF) [file pone.0232099.s010.pdf]
